# Dataset for interpreting the Circos figures used in the review of friction stir welding of titanium alloys

**DOI:** 10.1016/j.dib.2018.11.123

**Published:** 2018-11-29

**Authors:** Kapil Gangwar, M. Ramulu

**Affiliations:** University of Washington, 98195 Seattle, USA

**Keywords:** Circos data, Matrix visualization, Width of ribbons

## Abstract

In this paper we present the dataset used for plotting figure c (of graphical abstract) and figure 2 of the related article, “Friction stir welding of titanium alloys: A review” (Gangwar and Ramulu, 2018). For conventional descriptions, the majority of the data is either represented in forms of tables or graphs (2-dimensional, 3-dimensional and interactive). However, the presence of multiple variables and their interdependence require not only more dimensions but also a simple representation without clustering the information. This dataset includes the values of elements in the matrix used to plot Circos figures and describes their correspondence with the interrelation of columns and rows of matrix in the recurring figures in the article (Gangwar and Ramulu, 2018). This article also focuses on the width of ribbons in figure c and figure 2 that are connected via multiple alphabetic elements in rows and columns of the matrix.

## Specification table

**Table:** 

**Subject area**	Circos data visualization
**More specific subject area**	Representation of friction stir welding (FSW) variables
**Type of data**	Tables
**How data was acquired**	The correlation matrices of dimensions 13×1, and 24×24 were visualized with Circos plot
**Data format**	Raw, analyzed
**Experimental factors**	Interdependence of variables involved in FSW of titanium was closely studied
**Experimental features**	These variables were ranked between 0 and 16 according to their correlation with each other
**Data source location**	University of Washington, Seattle, Washington, 98115, USA
**Data accessibility**	The data is within this articles
**Related research article**	Gangwar, K., & Ramulu, M. (2018). Friction stir welding of titanium alloys: A review. Materials & Design, 141, 230–255 [1]

**Value of the data**

• The data presented in this article describes a method that can be used to understand the Circos figures in the related research article [1].
• While figures presented in the form of flow charts (to describe the interdependence of multiple variables) represent this state of the art technology (FSW) in greater details, we wanted to revise these figures [2] in rather aesthetic way to interact more effectively with the readers.
• Martin et al. [3] have created a visualization tool called Circos to facilitate the identification and analysis of similarities and differences arising from comparisons of genomes. While sequence alignments, hybridization arrays, genome mapping, and genotyping studies represent such datasets in a more conventional ways, Circos uses a circular ideogram layout to facilitate the display of relationships between pairs of positions by the use of ribbons, which encode the position, size, and orientation of related genomic elements.
• Furthermore, recent developments in Circos 2D track plots have been introduced as an additional RCircos package by Hongen et al. [4].
• Realizing the potential of such multi-dimensional data sets in genomic science and cancer research, we have taken a step in introducing the scientific community involved in manufacturing and variance analysis. Adaptability of Circos can further be realized with design of experiments where multiple variables are interdependent in optimizing the certain properties.

## 1 Data

Table 1, Table 2 represent the values of elements that were considered to plot Circos figures [1].

**Table 1:** Representation of data for figure c of graphical abstract in form of table.

**Data**	Titanium-Alloys-FSW
Dissimilar (Dissim.)	1
Fatigue	1
Material flow (Flow)	1
Future work (Future)	1
Microhardness (HV)	1
Metallurgical characteristics (Metall.)	1
Phase analysis (Phase)	1
Processing conditions (Process)	1
Residual stress (Residual)	1
Super plastic forming ability (SPF)	1
Evolution of temperatures (Temp.)	1
Tensile	1
Texture	1

**Table 2:** Representation of data for figure 2 in form of table.

**Data**	T1-ADV	T2-RET	Friction stir welding automation (FSW-A)	Tool	Argon gas flow (Ar-Flow)	Anvil	Taper	Thread	Cylindrical (Cyl.)	Mechanical characteristics (Mech.)	Wear	Design	Metall.	Temp.
Friction stir welded (FSWed)	16	16	8	8	8	8	0	0	0	0	0	0	0	0
Load	0	0	4	0	0	0	0	0	0	1	0	0	0	0
Displacement (Displac.)	0	0	4	0	0	0	0	0	0	1	0	0	0	0
Stiffness	0	0	4	0	0	0	0	0	0	1	0	0	0	0
Power	0	0	4	0	0	0	0	0	0	1	0	0	0	0
Design	0	0	0	4	0	0	2	2	2	0	0	0	0	0
Tool material (Mate.)	0	0	0	4	0	0	0	0	0	0	1	0	0	0
Wear	0	0	0	4	0	0	0	0	0	1	0	0	0	0
Tilt	4	4	0	4	0	0	0	0	0	0	0	0	0	0
Rotation per minute (rpm)	0	0	0	4	0	0	0	0	0	0	0	0	0	4
traverse	0	0	0	4	0	0	0	0	0	0	0	0	0	4
Strain rate (Strain-R.)	0	0	0	0	0	0	1	1	1	1	0	0	0	0
Metallurgical characteristics (Metall.)	16	16	0	0	0	0	0	0	0	1	1	0	0	0
Titanium alloy-1 on advancing side (T1-ADV)	0	0	0	0	0	0	0	0	0	16	0	0	0	0
Titanium alloy-2 on retreating side (T2-RET)	0	0	0	0	0	0	0	0	0	16	0	0	0	0
Evolving temperatures (Temp.)	0	0	0	0	4	4	0	0	0	0	1	1	4	0

The alphabetical elements of rows and columns (i.e. variables involved in FSW) are placed along the circumference of the circle in figure c and figure 2 in [1].

Depending on mutual interdependence of the alphabetical elements on each other, the length of arcs was determined. i.e. more dominating variable has the higher arc length.

The width of connecting ribbons from columns to rows and from rows to columns is determined by the value of the elements (as shown in Table 1, Table 2).

Connections from column to row are tightly connected (i.e. no gap between ribbon and arc) and from row to column are loosely connected (i.e. there is a gap between ribbon and arc).

## 2 Experimental design, materials and methods

While representing the matrix of multiple variables in Circos figures, we have carefully considered the effect of each variable on other variables. If two variables are isolated and have no connection with one another, that correlation is termed 0 in the matrix. Elements (1, 2, 4, 8, or 16) for interdependent variables, however, were defined based on their theoretical importance on processing, metallurgical and mechanical properties and structural performance of FSWed titanium alloys. Width of the connecting ribbons also varied from 1 (minimum) to 16 (maximum).

### 2.1 Design of figure c

Matrix representation of figure c in the graphical abstract [1] can be visualized as a matrix of 13×1for possible characteristics of FSW to be considered.

Since all the elements in Table 1 are 1, the ribbons of figure c are of equal width. The main theme of the article is titanium (Ti) alloys FSW, it has the highest arc length in figure c (of graphical abstract) of article [1].

### 2.2 Design of figure 2

The table used to generate figure 2 of related article [1].

## Funding sources

This work is part of a Ph.D. thesis of Kapil Gangwar and was supported by department of mechanical engineering at University of Washington located in Seattle, Washington, USA.
